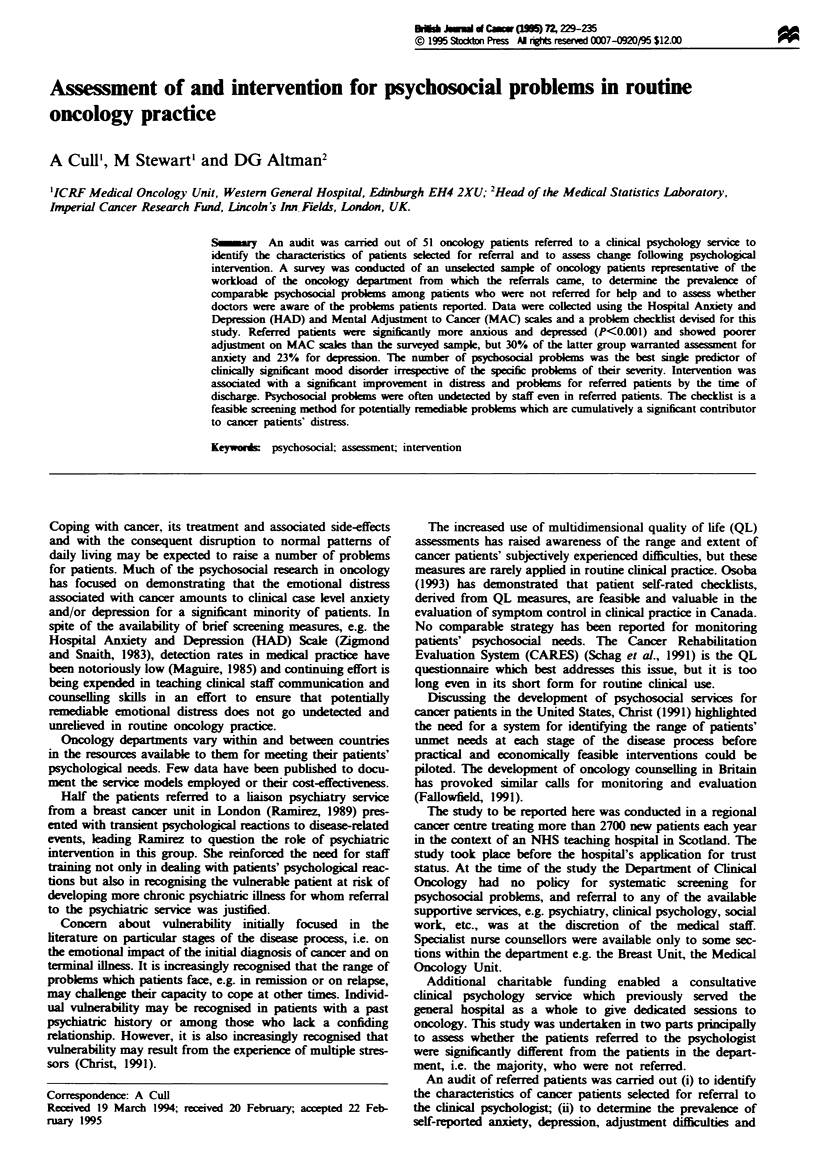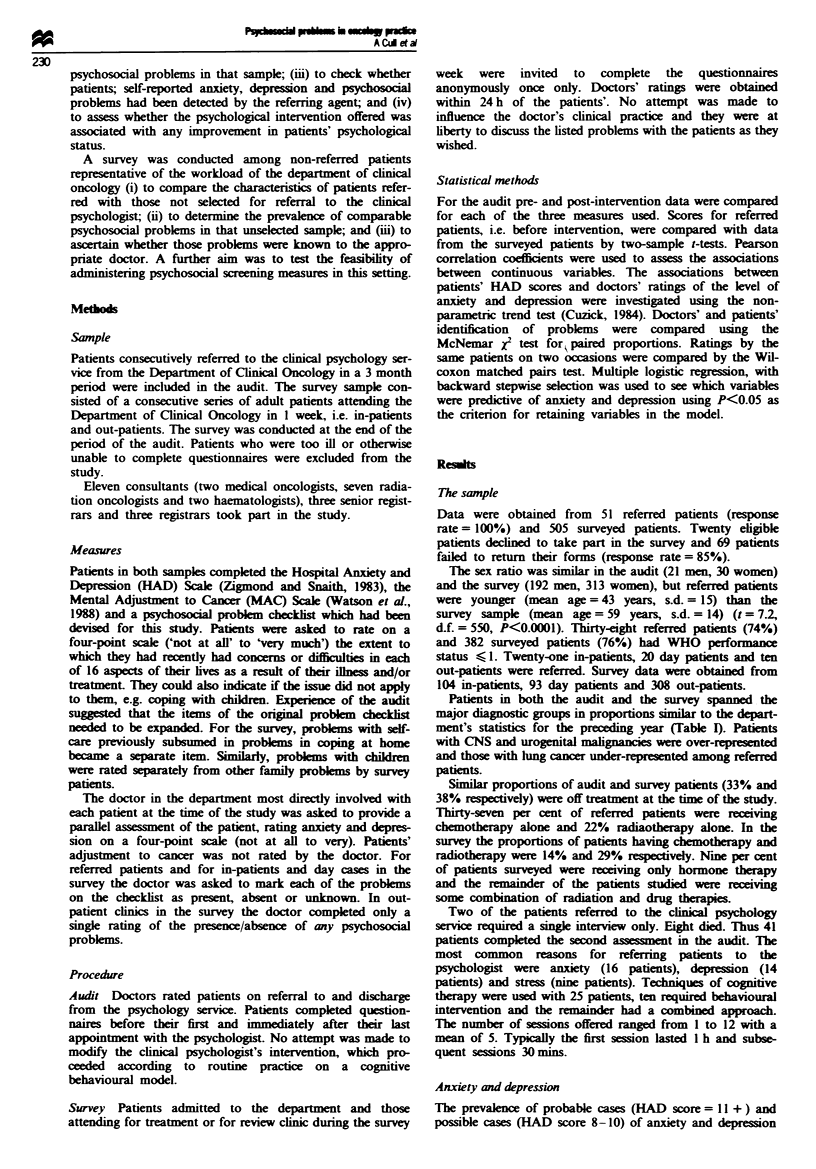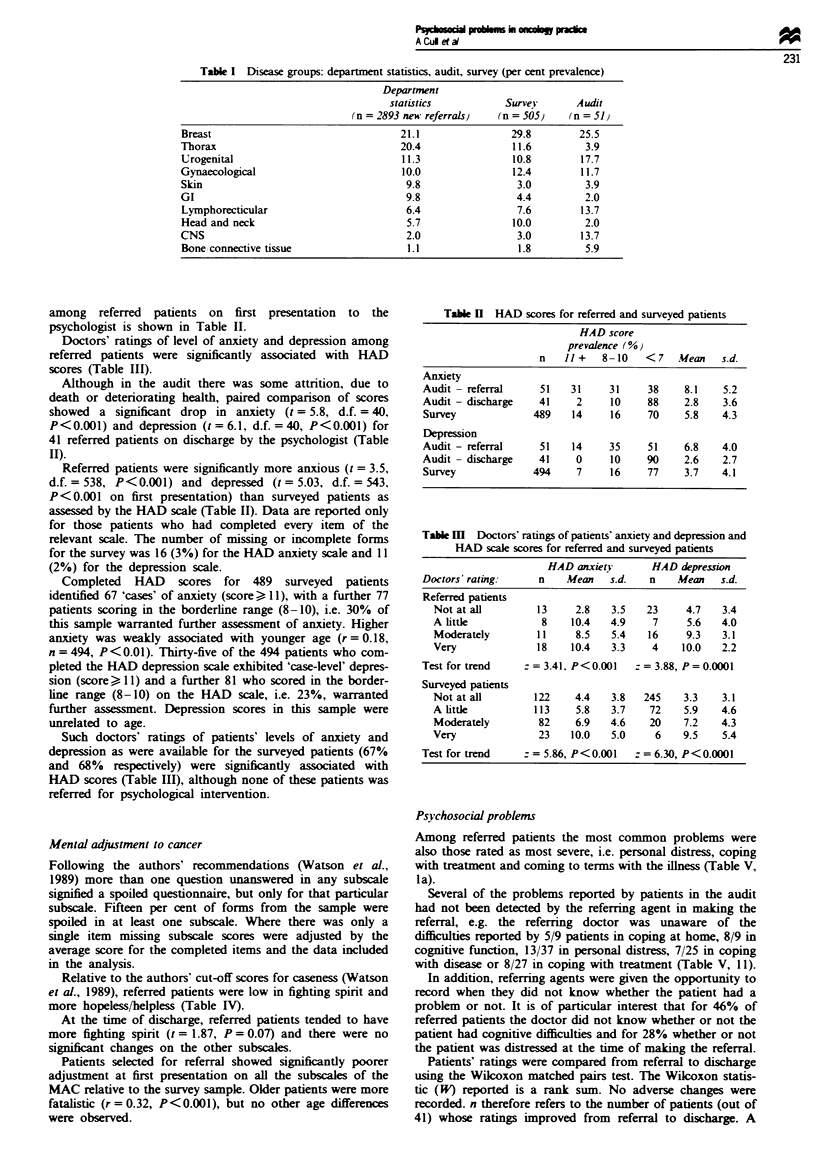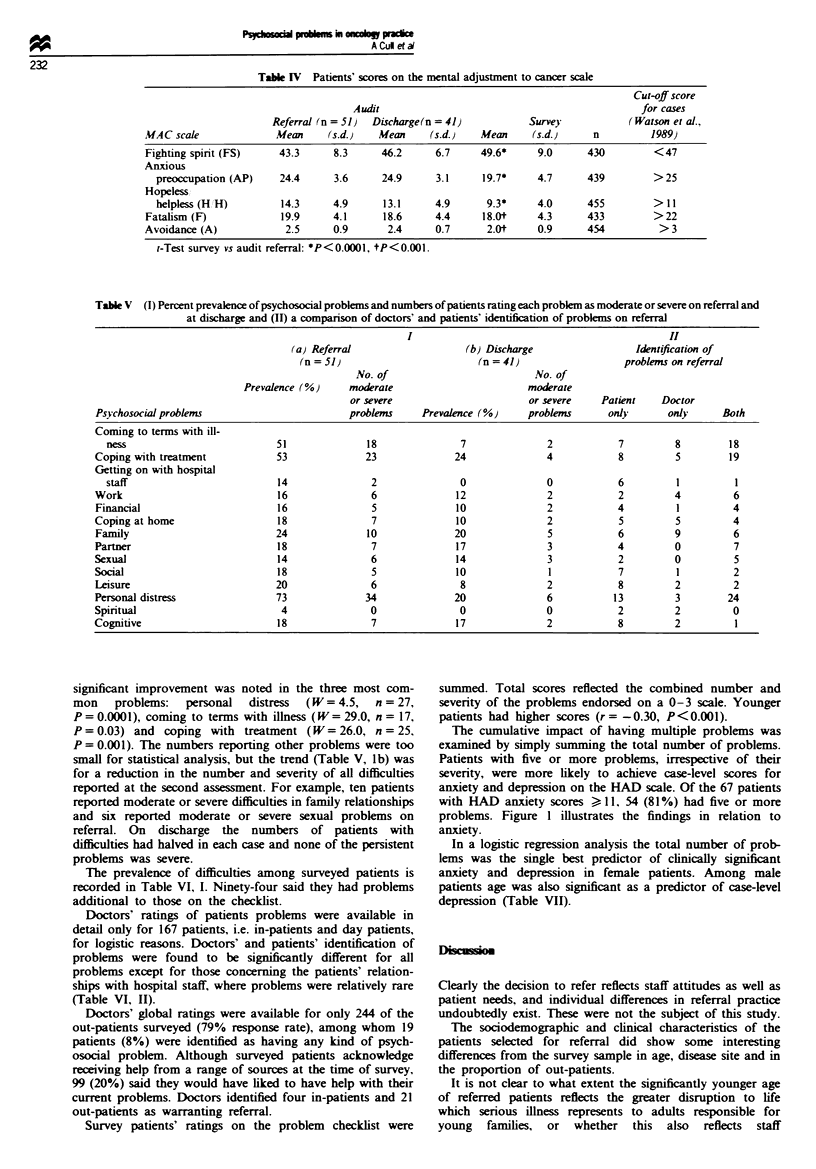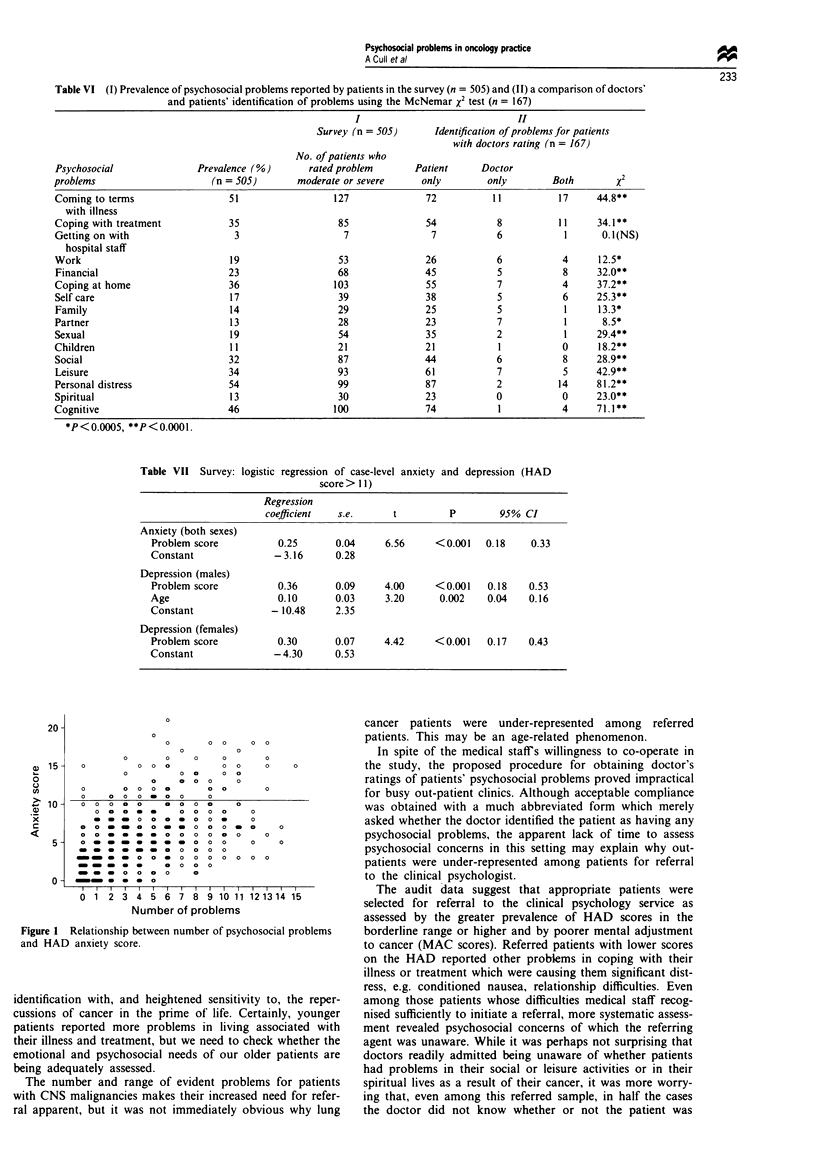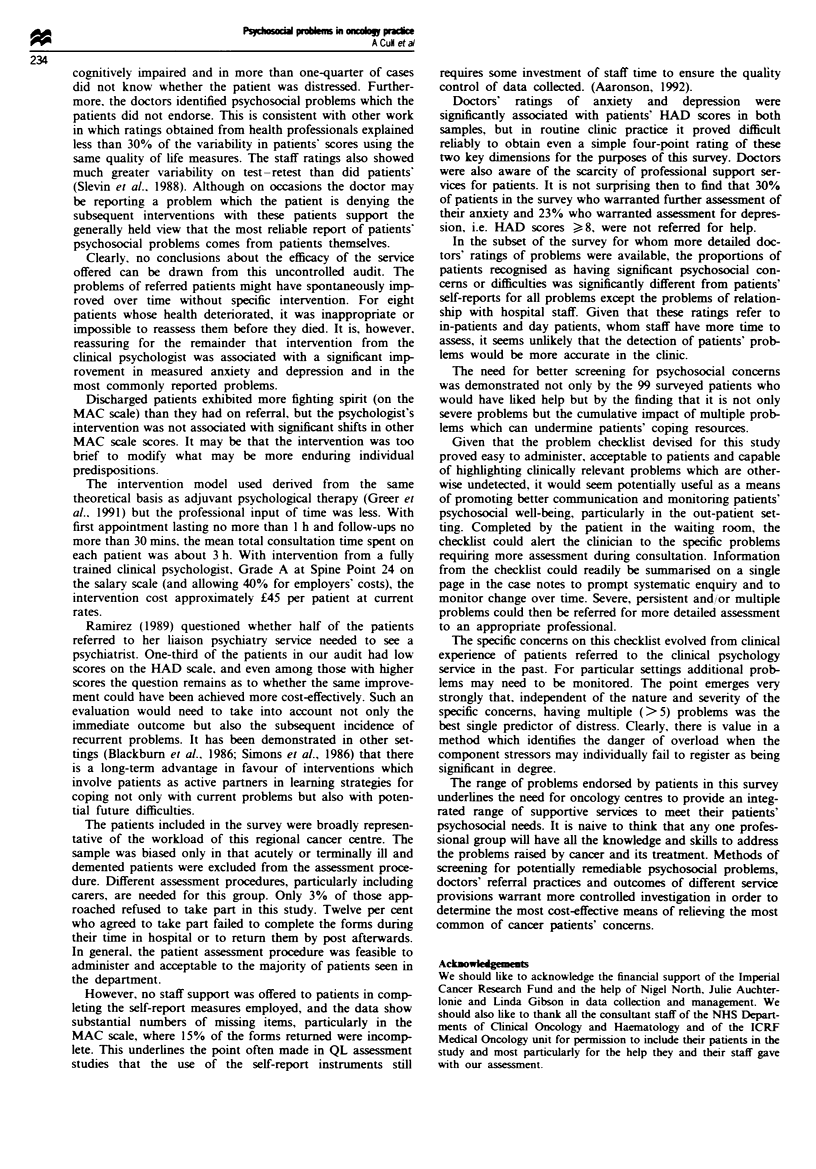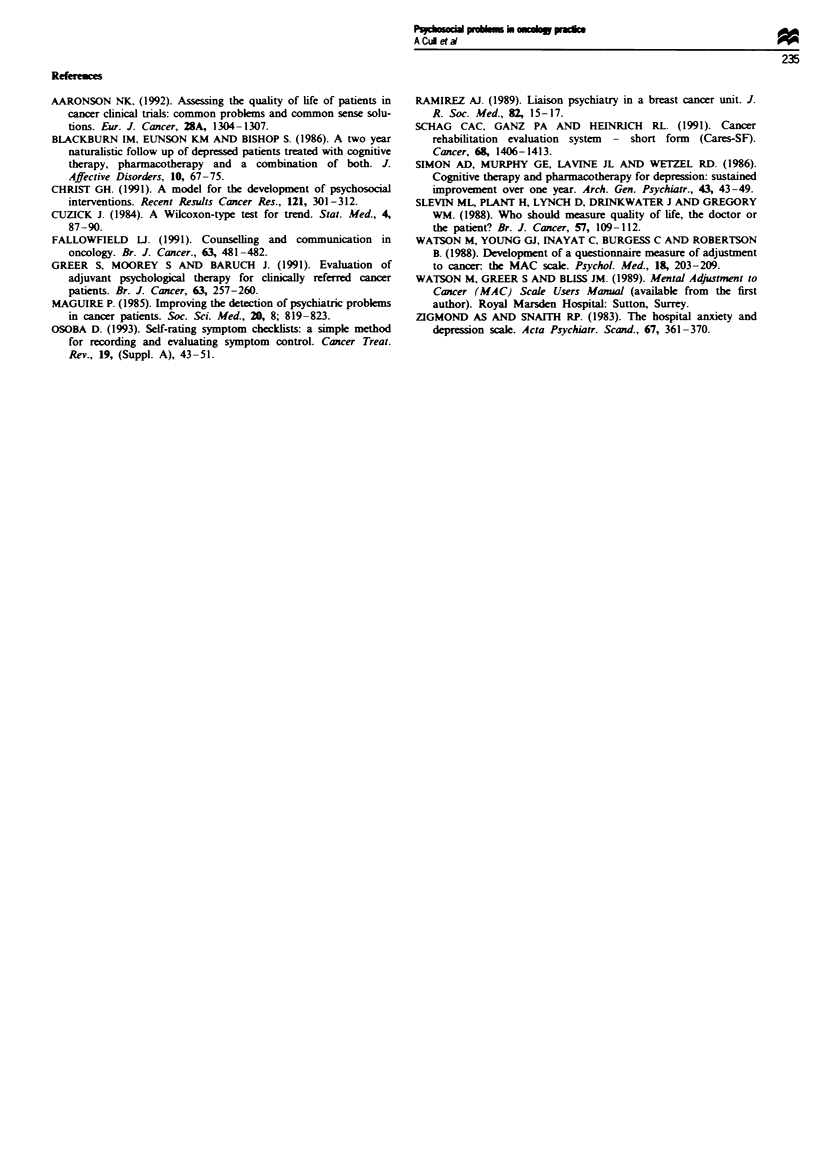# Assessment of and intervention for psychosocial problems in routine oncology practice.

**DOI:** 10.1038/bjc.1995.308

**Published:** 1995-07

**Authors:** A. Cull, M. Stewart, D. G. Altman

**Affiliations:** ICRF Medical Oncology Unit, Western General Hospital, Edinburgh, UK.

## Abstract

An audit was carried out of 51 oncology patients referred to a clinical psychology service to identify the characteristics of patients selected for referral and to assess change following psychological intervention. A survey was conducted of an unselected sample of oncology patients representative of the workload of the oncology department from which the referrals came, to determine the prevalence of comparable psychosocial problems among patients who were not referred for help and to assess whether doctors were aware of the problems patients reported. Data were collected using the Hospital Anxiety and Depression (HAD) and Mental Adjustment to Cancer (MAC) scales and a problem checklist devised for this study. Referred patients were significantly more anxious and depressed (P < 0.001) and showed poorer adjustment on MAC scales than the surveyed sample, but 30% of the latter group warranted assessment for anxiety and 23% for depression. The number of psychosocial problems of their severity. Intervention was clinically significant mood disorder irrespective of the specific problems of their severity. Intervention was associated with a significant improvement in distress and problems for referred patients by the time of discharge. Psychosocial problems were often undetected by staff even in referred patients. The checklist is a feasible screening method for potentially remediable problems which are cumulatively a significant contributor to cancer patients' distress.


British Journal of Cancer (1995) 72, 229-235

© 1995 Stockton Press All rights reserved 0007-0920/95 $12.00

### Assessment of and intervention for psychosocial problems in routine oncology practice

A Cull[1], M Stewart[1] and DG Altman[2]

[1]*ICRF Medical Oncology Unit, Western General Hospital, Edinburgh EH4 2XU;* [2]*Head of the Medical Statistics Laboratory, Imperial Cancer Research Fund, Lincoln's Inn Fields, London, UK.*

**Summary** An audit was carried out of 51 oncology patients referred to a clinical psychology service to identify the characteristics of patients selected for referral and to assess change following psychological intervention. A survey was conducted of an unselected sample of oncology patients representative of the workload of the oncology department from which the referrals came, to determine the prevalence of comparable psychosocial problems among patients who were not referred for help and to assess whether doctors were aware of the problems patients reported. Data were collected using the Hospital Anxiety and Depression (HAD) and Mental Adjustment to Cancer (MAC) scales and a problem checklist devised for this study. Referred patients were significantly more anxious and depressed ($P<0.001$) and showed poorer adjustment on MAC scales than the surveyed sample, but 30% of the latter group warranted assessment for anxiety and 23% for depression. The number of psychosocial problems was the best single predictor of clinically significant mood disorder irrespective of the specific problems of their severity. Intervention was associated with a significant improvement in distress and problems for referred patients by the time of discharge. Psychosocial problems were often undetected by staff even in referred patients. The checklist is a feasible screening method for potentially remediable problems which are cumulatively a significant contributor to cancer patients' distress.

**Keywords:** psychosocial; assessment; intervention

Coping with cancer, its treatment and associated side-effects and with the consequent disruption to normal patterns of daily living may be expected to raise a number of problems for patients. Much of the psychosocial research in oncology has focused on demonstrating that the emotional distress associated with cancer amounts to clinical case level anxiety and/or depression for a significant minority of patients. In spite of the availability of brief screening measures, e.g. the Hospital Anxiety and Depression (HAD) Scale (Zigmond and Snaith, 1983), detection rates in medical practice have been notoriously low (Maguire, 1985) and continuing effort is being expended in teaching clinical staff communication and counselling skills in an effort to ensure that potentially remediable emotional distress does not go undetected and unrelieved in routine oncology practice.

Oncology departments vary within and between countries in the resources available to them for meeting their patients' psychological needs. Few data have been published to document the service models employed or their cost-effectiveness.

Half the patients referred to a liaison psychiatry service from a breast cancer unit in London (Ramirez, 1989) presented with transient psychological reactions to disease-related events, leading Ramirez to question the role of psychiatric intervention in this group. She reinforced the need for staff training not only in dealing with patients' psychological reactions but also in recognising the vulnerable patient at risk of developing more chronic psychiatric illness for whom referral to the psychiatric service was justified.

Concern about vulnerability initially focused in the literature on particular stages of the disease process, i.e. on the emotional impact of the initial diagnosis of cancer and on terminal illness. It is increasingly recognised that the range of problems which patients face, e.g. in remission or on relapse, may challenge their capacity to cope at other times. Individual vulnerability may be recognised in patients with a past psychiatric history or among those who lack a confiding relationship. However, it is also increasingly recognised that vulnerability may result from the experience of multiple stressors (Christ, 1991).

Correspondence: A Cull
Received 19 March 1994; received 20 February; accepted 22 February 1995

The increased use of multidimensional quality of life (QL) assessments has raised awareness of the range and extent of cancer patients' subjectively experienced difficulties, but these measures are rarely applied in routine clinical practice. Osoba (1993) has demonstrated that patient self-rated checklists, derived from QL measures, are feasible and valuable in the evaluation of symptom control in clinical practice in Canada. No comparable strategy has been reported for monitoring patients' psychosocial needs. The Cancer Rehabilitation Evaluation System (CARES) (Schag *et al.*, 1991) is the QL questionnaire which best addresses this issue, but it is too long even in its short form for routine clinical use.

Discussing the development of psychosocial services for cancer patients in the United States, Christ (1991) highlighted the need for a system for identifying the range of patients' unmet needs at each stage of the disease process before practical and economically feasible interventions could be piloted. The development of oncology counselling in Britain has provoked similar calls for monitoring and evaluation (Fallowfield, 1991).

The study to be reported here was conducted in a regional cancer centre treating more than 2700 new patients each year in the context of an NHS teaching hospital in Scotland. The study took place before the hospital's application for trust status. At the time of the study the Department of Clinical Oncology had no policy for systematic screening for psychosocial problems, and referral to any of the available supportive services, e.g. psychiatry, clinical psychology, social work, etc., was at the discretion of the medical staff. Specialist nurse counsellors were available only to some sections within the department e.g. the Breast Unit, the Medical Oncology Unit.

Additional charitable funding enabled a consultative clinical psychology service which previously served the general hospital as a whole to give dedicated sessions to oncology. This study was undertaken in two parts principally to assess whether the patients referred to the psychologist were significantly different from the patients in the department, i.e. the majority, who were not referred.

An audit of referred patients was carried out (i) to identify the characteristics of cancer patients selected for referral to the clinical psychologist; (ii) to determine the prevalence of self-reported anxiety, depression, adjustment difficulties and psychosocial problems in that sample; (iii) to check whether patients; self-reported anxiety, depression and psychosocial problems had been detected by the referring agent; and (iv) to assess whether the psychological intervention offered was associated with any improvement in patients' psychological status.

A survey was conducted among non-referred patients representative of the workload of the department of clinical oncology (i) to compare the characteristics of patients referred with those not selected for referral to the clinical psychologist; (ii) to determine the prevalence of comparable psychosocial problems in that unselected sample; and (iii) to ascertain whether those problems were known to the appropriate doctor. A further aim was to test the feasibility of administering psychosocial screening measures in this setting.

### Methods

### *Sample*

Patients consecutively referred to the clinical psychology service from the Department of Clinical Oncology in a 3 month period were included in the audit. The survey sample consisted of a consecutive series of adult patients attending the Department of Clinical Oncology in 1 week, i.e. in-patients and out-patients. The survey was conducted at the end of the period of the audit. Patients who were too ill or otherwise unable to complete questionnaires were excluded from the study.

Eleven consultants (two medical oncologists, seven radiation oncologists and two haematologists), three senior registrars and three registrars took part in the study.

### *Measures*

Patients in both samples completed the Hospital Anxiety and Depression (HAD) Scale (Zigmond and Snaith, 1983), the Mental Adjustment to Cancer (MAC) Scale (Watson *et al.*, 1988) and a psychosocial problem checklist which had been devised for this study. Patients were asked to rate on a four-point scale ('not at all' to 'very much') the extent to which they had recently had concerns or difficulties in each of 16 aspects of their lives as a result of their illness and/or treatment. They could also indicate if the issue did not apply to them, e.g. coping with children. Experience of the audit suggested that the items of the original problem checklist needed to be expanded. For the survey, problems with self-care previously subsumed in problems in coping at home became a separate item. Similarly, problems with children were rated separately from other family problems by survey patients.

The doctor in the department most directly involved with each patient at the time of the study was asked to provide a parallel assessment of the patient, rating anxiety and depression on a four-point scale (not at all to very). Patients' adjustment to cancer was not rated by the doctor. For referred patients and for in-patients and day cases in the survey the doctor was asked to mark each of the problems on the checklist as present, absent or unknown. In out-patient clinics in the survey the doctor completed only a single rating of the presence/absence of *any* psychosocial problems.

### *Procedure*

*Audit* Doctors rated patients on referral to and discharge from the psychology service. Patients completed questionnaires before their first and immediately after their last appointment with the psychologist. No attempt was made to modify the clinical psychologist's intervention, which proceeded according to routine practice on a cognitive behavioural model.

*Survey* Patients admitted to the department and those attending for treatment or for review clinic during the survey week were invited to complete the questionnaires anonymously once only. Doctors' ratings were obtained within 24 h of the patients'. No attempt was made to influence the doctor's clinical practice and they were at liberty to discuss the listed problems with the patients as they wished.

### *Statistical methods*

For the audit pre- and post-intervention data were compared for each of the three measures used. Scores for referred patients, i.e. before intervention, were compared with data from the surveyed patients by two-sample *t*-tests. Pearson correlation coefficients were used to assess the associations between continuous variables. The associations between patients' HAD scores and doctors' ratings of the level of anxiety and depression were investigated using the non-parametric trend test (Cuzick, 1984). Doctors' and patients' identification of problems were compared using the McNemar $\chi^2$ test for paired proportions. Ratings by the same patients on two occasions were compared by the Wilcoxon matched pairs test. Multiple logistic regression, with backward stepwise selection was used to see which variables were predictive of anxiety and depression using $P<0.05$ as the criterion for retaining variables in the model.

### Results

### *The sample*

Data were obtained from 51 referred patients (response rate = 100%) and 505 surveyed patients. Twenty eligible patients declined to take part in the survey and 69 patients failed to return their forms (response rate = 85%).

The sex ratio was similar in the audit (21 men, 30 women) and the survey (192 men, 313 women), but referred patients were younger (mean age = 43 years, s.d. = 15) than the survey sample (mean age = 59 years, s.d. = 14) ($t = 7.2$, d.f. = 550, $P<0.0001$). Thirty-eight referred patients (74%) and 382 surveyed patients (76%) had WHO performance status $\leqslant 1$. Twenty-one in-patients, 20 day patients and ten out-patients were referred. Survey data were obtained from 104 in-patients, 93 day patients and 308 out-patients.

Patients in both the audit and the survey spanned the major diagnostic groups in proportions similar to the department's statistics for the preceding year (Table I). Patients with CNS and urogenital malignancies were over-represented and those with lung cancer under-represented among referred patients.

Similar proportions of audit and survey patients (33% and 38% respectively) were off treatment at the time of the study. Thirty-seven per cent of referred patients were receiving chemotherapy alone and 22% radiaotherapy alone. In the survey the proportions of patients having chemotherapy and radiotherapy were 14% and 29% respectively. Nine per cent of patients surveyed were receiving only hormone therapy and the remainder of the patients studied were receiving some combination of radiation and drug therapies.

Two of the patients referred to the clinical psychology service required a single interview only. Eight died. Thus 41 patients completed the second assessment in the audit. The most common reasons for referring patients to the psychologist were anxiety (16 patients), depression (14 patients) and stress (nine patients). Techniques of cognitive therapy were used with 25 patients, ten required behavioural intervention and the remainder had a combined approach. The number of sessions offered ranged from 1 to 12 with a mean of 5. Typically the first session lasted 1 h and subsequent sessions 30 mins.

### *Anxiety and depression*

The prevalence of probable cases (HAD score = 11 + ) and possible cases (HAD score 8–10) of anxiety and depression

**Table I** Disease groups: department statistics, audit, survey (per cent prevalence)

| | *Department statistics* (n = *2893 new referrals*) | *Survey* (n = *505*) | *Audit* (n = *51*) |
|---|---|---|---|
| Breast | 21.1 | 29.8 | 25.5 |
| Thorax | 20.4 | 11.6 | 3.9 |
| Urogenital | 11.3 | 10.8 | 17.7 |
| Gynaecological | 10.0 | 12.4 | 11.7 |
| Skin | 9.8 | 3.0 | 3.9 |
| GI | 9.8 | 4.4 | 2.0 |
| Lymphorecticular | 6.4 | 7.6 | 13.7 |
| Head and neck | 5.7 | 10.0 | 2.0 |
| CNS | 2.0 | 3.0 | 13.7 |
| Bone/connective tissue | 1.1 | 1.8 | 5.9 |

among referred patients on first presentation to the psychologist is shown in Table II.

Doctors' ratings of level of anxiety and depression among referred patients were significantly associated with HAD scores (Table III).

Although in the audit there was some attrition, due to death or deteriorating health, paired comparison of scores showed a significant drop in anxiety ($t = 5.8$, d.f. = 40, $P < 0.001$) and depression ($t = 6.1$, d.f. = 40, $P < 0.001$) for 41 referred patients on discharge by the psychologist (Table II).

Referred patients were significantly more anxious ($t = 3.5$, d.f. = 538, $P < 0.001$) and depressed ($t = 5.03$, d.f. = 543, $P < 0.001$ on first presentation) than surveyed patients as assessed by the HAD scale (Table II). Data are reported only for those patients who had completed every item of the relevant scale. The number of missing or incomplete forms for the survey was 16 (3%) for the HAD anxiety scale and 11 (2%) for the depression scale.

Completed HAD scores for 489 surveyed patients identified 67 'cases' of anxiety (score $\geqslant 11$), with a further 77 patients scoring in the borderline range (8–10), i.e. 30% of this sample warranted further assessment of anxiety. Higher anxiety was weakly associated with younger age ($r = 0.18$, $n = 494$, $P < 0.01$). Thirty-five of the 494 patients who completed the HAD depression scale exhibited 'case-level' depression (score $\geqslant 11$) and a further 81 who scored in the borderline range (8–10) on the HAD scale, i.e. 23%, warranted further assessment. Depression scores in this sample were unrelated to age.

Such doctors' ratings of patients' levels of anxiety and depression as were available for the surveyed patients (67% and 68% respectively) were significantly associated with HAD scores (Table III), although none of these patients was referred for psychological intervention.

### *Mental adjustment to cancer*

Following the authors' recommendations (Watson *et al.*, 1989) more than one question unanswered in any subscale signified a spoiled questionnaire, but only for that particular subscale. Fifteen per cent of forms from the sample were spoiled in at least one subscale. Where there was only a single item missing subscale scores were adjusted by the average score for the completed items and the data included in the analysis.

Relative to the authors' cut-off scores for caseness (Watson *et al.*, 1989), referred patients were low in fighting spirit and more hopeless/helpless (Table IV).

At the time of discharge, referred patients tended to have more fighting spirit ($t = 1.87$, $P = 0.07$) and there were no significant changes on the other subscales.

Patients selected for referral showed significantly poorer adjustment at first presentation on all the subscales of the MAC relative to the survey sample. Older patients were more fatalistic ($r = 0.32$, $P < 0.001$), but no other age differences were observed.

**Table II** HAD scores for referred and surveyed patients

| | n | *HAD score prevalence (%)* *11+* | *8–10* | *<7* | *Mean* | *s.d.* |
|---|---|---|---|---|---|---|
| Anxiety | | | | | | |
| Audit – referral | 51 | 31 | 31 | 38 | 8.1 | 5.2 |
| Audit – discharge | 41 | 2 | 10 | 88 | 2.8 | 3.6 |
| Survey | 489 | 14 | 16 | 70 | 5.8 | 4.3 |
| Depression | | | | | | |
| Audit – referral | 51 | 14 | 35 | 51 | 6.8 | 4.0 |
| Audit – discharge | 41 | 0 | 10 | 90 | 2.6 | 2.7 |
| Survey | 494 | 7 | 16 | 77 | 3.7 | 4.1 |

**Table III** Doctors' ratings of patients' anxiety and depression and HAD scale scores for referred and surveyed patients

| | *HAD anxiety* | | | *HAD depression* | | |
|---|---|---|---|---|---|---|
| *Doctors' rating:* | n | *Mean* | *s.d.* | n | *Mean* | *s.d.* |
| Referred patients | | | | | | |
| Not at all | 13 | 2.8 | 3.5 | 23 | 4.7 | 3.4 |
| A little | 8 | 10.4 | 4.9 | 7 | 5.6 | 4.0 |
| Moderately | 11 | 8.5 | 5.4 | 16 | 9.3 | 3.1 |
| Very | 18 | 10.4 | 3.3 | 4 | 10.0 | 2.2 |
| Test for trend | $z = 3.41$, $P < 0.001$ | | | $z = 3.88$, $P = 0.0001$ | | |
| Surveyed patients | | | | | | |
| Not at all | 122 | 4.4 | 3.8 | 245 | 3.3 | 3.1 |
| A little | 113 | 5.8 | 3.7 | 72 | 5.9 | 4.6 |
| Moderately | 82 | 6.9 | 4.6 | 20 | 7.2 | 4.3 |
| Very | 23 | 10.0 | 5.0 | 6 | 9.5 | 5.4 |
| Test for trend | $z = 5.86$, $P < 0.001$ | | | $z = 6.30$, $P < 0.0001$ | | |

### *Psychosocial problems*

Among referred patients the most common problems were also those rated as most severe, i.e. personal distress, coping with treatment and coming to terms with the illness (Table V, 1a).

Several of the problems reported by patients in the audit had not been detected by the referring agent in making the referral, e.g. the referring doctor was unaware of the difficulties reported by 5/9 patients in coping at home, 8/9 in cognitive function, 13/37 in personal distress, 7/25 in coping with disease or 8/27 in coping with treatment (Table V, 11).

In addition, referring agents were given the opportunity to record when they did not know whether the patient had a problem or not. It is of particular interest that for 46% of referred patients the doctor did not know whether or not the patient had cognitive difficulties and for 28% whether or not the patient was distressed at the time of making the referral.

Patients' ratings were compared from referral to discharge using the Wilcoxon matched pairs test. The Wilcoxon statistic (*W*) reported is a rank sum. No adverse changes were recorded. *n* therefore refers to the number of patients (out of 41) whose ratings improved from referral to discharge. A

**Table IV** Patients' scores on the mental adjustment to cancer scale

| MAC scale | *Audit* *Referral* (n = 51) *Mean* | (*s.d.*) | *Discharge* (n = 41) *Mean* | (*s.d.*) | *Survey* *Mean* | (*s.d.*) | n | *Cut-off score for cases* (*Watson et al.*, *1989*) |
|---|---|---|---|---|---|---|---|---|
| Fighting spirit (FS) | 43.3 | 8.3 | 46.2 | 6.7 | 49.6* | 9.0 | 430 | <47 |
| Anxious preoccupation (AP) | 24.4 | 3.6 | 24.9 | 3.1 | 19.7* | 4.7 | 439 | >25 |
| Hopeless/helpless (H/H) | 14.3 | 4.9 | 13.1 | 4.9 | 9.3* | 4.0 | 455 | >11 |
| Fatalism (F) | 19.9 | 4.1 | 18.6 | 4.4 | 18.0† | 4.3 | 433 | >22 |
| Avoidance (A) | 2.5 | 0.9 | 2.4 | 0.7 | 2.0† | 0.9 | 454 | >3 |

$t$-Test survey *vs* audit referral: *$P<0.0001$, †$P<0.001$.

**Table V** (I) Percent prevalence of psychosocial problems and numbers of patients rating each problem as moderate or severe on referral and at discharge and (II) a comparison of doctors' and patients' identification of problems on referral

| *Psychosocial problems* | I (*a*) *Referral* (n = *51*) *Prevalence* (%) | *No. of moderate or severe problems* | (*b*) *Discharge* (n = *41*) *Prevalence* (%) | *No. of moderate or severe problems* | II *Identification of problems on referral* *Patient only* | *Doctor only* | *Both* |
|---|---|---|---|---|---|---|---|
| Coming to terms with illness | 51 | 18 | 7 | 2 | 7 | 8 | 18 |
| Coping with treatment | 53 | 23 | 24 | 4 | 8 | 5 | 19 |
| Getting on with hospital staff | 14 | 2 | 0 | 0 | 6 | 1 | 1 |
| Work | 16 | 6 | 12 | 2 | 2 | 4 | 6 |
| Financial | 16 | 5 | 10 | 2 | 4 | 1 | 4 |
| Coping at home | 18 | 7 | 10 | 2 | 5 | 5 | 4 |
| Family | 24 | 10 | 20 | 5 | 6 | 9 | 6 |
| Partner | 18 | 7 | 17 | 3 | 4 | 0 | 7 |
| Sexual | 14 | 6 | 14 | 3 | 2 | 0 | 5 |
| Social | 18 | 5 | 10 | 1 | 7 | 1 | 2 |
| Leisure | 20 | 6 | 8 | 2 | 8 | 2 | 2 |
| Personal distress | 73 | 34 | 20 | 6 | 13 | 3 | 24 |
| Spiritual | 4 | 0 | 0 | 0 | 2 | 2 | 0 |
| Cognitive | 18 | 7 | 17 | 2 | 8 | 2 | 1 |

significant improvement was noted in the three most common problems: personal distress ($W=4.5$, $n=27$, $P=0.0001$), coming to terms with illness ($W=29.0$, $n=17$, $P=0.03$) and coping with treatment ($W=26.0$, $n=25$, $P=0.001$). The numbers reporting other problems were too small for statistical analysis, but the trend (Table V, 1b) was for a reduction in the number and severity of all difficulties reported at the second assessment. For example, ten patients reported moderate or severe difficulties in family relationships and six reported moderate or severe sexual problems on referral. On discharge the numbers of patients with difficulties had halved in each case and none of the persistent problems was severe.

The prevalence of difficulties among surveyed patients is recorded in Table VI, I. Ninety-four said they had problems additional to those on the checklist.

Doctors' ratings of patients problems were available in detail only for 167 patients, i.e. in-patients and day patients, for logistic reasons. Doctors' and patients' identification of problems were found to be significantly different for all problems except for those concerning the patients' relationships with hospital staff, where problems were relatively rare (Table VI, II).

Doctors' global ratings were available for only 244 of the out-patients surveyed (79% response rate), among whom 19 patients (8%) were identified as having any kind of psychosocial problem. Although surveyed patients acknowledge receiving help from a range of sources at the time of survey, 99 (20%) said they would have liked to have help with their current problems. Doctors identified four in-patients and 21 out-patients as warranting referral.

Survey patients' ratings on the problem checklist were summed. Total scores reflected the combined number and severity of the problems endorsed on a 0–3 scale. Younger patients had higher scores ($r=-0.30$, $P<0.001$).

The cumulative impact of having multiple problems was examined by simply summing the total number of problems. Patients with five or more problems, irrespective of their severity, were more likely to achieve case-level scores for anxiety and depression on the HAD scale. Of the 67 patients with HAD anxiety scores $\geqslant 11$, 54 (81%) had five or more problems. Figure 1 illustrates the findings in relation to anxiety.

In a logistic regression analysis the total number of problems was the single best predictor of clinically significant anxiety and depression in female patients. Among male patients age was also significant as a predictor of case-level depression (Table VII).

### Discussion

Clearly the decision to refer reflects staff attitudes as well as patient needs, and individual differences in referral practice undoubtedly exist. These were not the subject of this study.

The sociodemographic and clinical characteristics of the patients selected for referral did show some interesting differences from the survey sample in age, disease site and in the proportion of out-patients.

It is not clear to what extent the significantly younger age of referred patients reflects the greater disruption to life which serious illness represents to adults responsible for young families, or whether this also reflects staff

**Table VI** (I) Prevalence of psychosocial problems reported by patients in the survey ($n$ = 505) and (II) a comparison of doctors' and patients' identification of problems using the McNemar $\chi^2$ test ($n$ = 167)

| | | *I* Survey (n = 505) | *II* Identification of problems for patients with doctors rating (n = 167) | | | |
|---|---|---|---|---|---|---|
| *Psychosocial problems* | *Prevalence (%) (n = 505)* | *No. of patients who rated problem moderate or severe* | *Patient only* | *Doctor only* | *Both* | $\chi^2$ |
| Coming to terms with illness | 51 | 127 | 72 | 11 | 17 | 44.8** |
| Coping with treatment | 35 | 85 | 54 | 8 | 11 | 34.1** |
| Getting on with hospital staff | 3 | 7 | 7 | 6 | 1 | 0.1(NS) |
| Work | 19 | 53 | 26 | 6 | 4 | 12.5* |
| Financial | 23 | 68 | 45 | 5 | 8 | 32.0** |
| Coping at home | 36 | 103 | 55 | 7 | 4 | 37.2** |
| Self care | 17 | 39 | 38 | 5 | 6 | 25.3** |
| Family | 14 | 29 | 25 | 5 | 1 | 13.3* |
| Partner | 13 | 28 | 23 | 7 | 1 | 8.5* |
| Sexual | 19 | 54 | 35 | 2 | 1 | 29.4** |
| Children | 11 | 21 | 21 | 1 | 0 | 18.2** |
| Social | 32 | 87 | 44 | 6 | 8 | 28.9** |
| Leisure | 34 | 93 | 61 | 7 | 5 | 42.9** |
| Personal distress | 54 | 99 | 87 | 2 | 14 | 81.2** |
| Spiritual | 13 | 30 | 23 | 0 | 0 | 23.0** |
| Cognitive | 46 | 100 | 74 | 1 | 4 | 71.1** |

*$P<0.0005$, **$P<0.0001$.

**Table VII** Survey: logistic regression of case-level anxiety and depression (HAD score > 11)

| | *Regression coefficient* | *s.e.* | t | P | *95% CI* | |
|---|---|---|---|---|---|---|
| Anxiety (both sexes) | | | | | | |
| Problem score | 0.25 | 0.04 | 6.56 | <0.001 | 0.18 | 0.33 |
| Constant | −3.16 | 0.28 | | | | |
| Depression (males) | | | | | | |
| Problem score | 0.36 | 0.09 | 4.00 | <0.001 | 0.18 | 0.53 |
| Age | 0.10 | 0.03 | 3.20 | 0.002 | 0.04 | 0.16 |
| Constant | −10.48 | 2.35 | | | | |
| Depression (females) | | | | | | |
| Problem score | 0.30 | 0.07 | 4.42 | <0.001 | 0.17 | 0.43 |
| Constant | −4.30 | 0.53 | | | | |

**Figure 1** Relationship between number of psychosocial problems and HAD anxiety score.

identification with, and heightened sensitivity to, the repercussions of cancer in the prime of life. Certainly, younger patients reported more problems in living associated with their illness and treatment, but we need to check whether the emotional and psychosocial needs of our older patients are being adequately assessed.

The number and range of evident problems for patients with CNS malignancies makes their increased need for referral apparent, but it was not immediately obvious why lung cancer patients were under-represented among referred patients. This may be an age-related phenomenon.

In spite of the medical staff's willingness to co-operate in the study, the proposed procedure for obtaining doctor's ratings of patients' psychosocial problems proved impractical for busy out-patient clinics. Although acceptable compliance was obtained with a much abbreviated form which merely asked whether the doctor identified the patient as having any psychosocial problems, the apparent lack of time to assess psychosocial concerns in this setting may explain why out-patients were under-represented among patients for referral to the clinical psychologist.

The audit data suggest that appropriate patients were selected for referral to the clinical psychology service as assessed by the greater prevalence of HAD scores in the borderline range or higher and by poorer mental adjustment to cancer (MAC scores). Referred patients with lower scores on the HAD reported other problems in coping with their illness or treatment which were causing them significant distress, e.g. conditioned nausea, relationship difficulties. Even among those patients whose difficulties medical staff recognised sufficiently to initiate a referral, more systematic assessment revealed psychosocial concerns of which the referring agent was unaware. While it was perhaps not surprising that doctors readily admitted being unaware of whether patients had problems in their social or leisure activities or in their spiritual lives as a result of their cancer, it was more worrying that, even among this referred sample, in half the cases the doctor did not know whether or not the patient was cognitively impaired and in more than one-quarter of cases did not know whether the patient was distressed. Furthermore, the doctors identified psychosocial problems which the patients did not endorse. This is consistent with other work in which ratings obtained from health professionals explained less than 30% of the variability in patients' scores using the same quality of life measures. The staff ratings also showed much greater variability on test–retest than did patients' (Slevin *et al.*, 1988). Although on occasions the doctor may be reporting a problem which the patient is denying the subsequent interventions with these patients support the generally held view that the most reliable report of patients' psychosocial problems comes from patients themselves.

Clearly, no conclusions about the efficacy of the service offered can be drawn from this uncontrolled audit. The problems of referred patients might have spontaneously improved over time without specific intervention. For eight patients whose health deteriorated, it was inappropriate or impossible to reassess them before they died. It is, however, reassuring for the remainder that intervention from the clinical psychologist was associated with a significant improvement in measured anxiety and depression and in the most commonly reported problems.

Discharged patients exhibited more fighting spirit (on the MAC scale) than they had on referral, but the psychologist's intervention was not associated with significant shifts in other MAC scale scores. It may be that the intervention was too brief to modify what may be more enduring individual predispositions.

The intervention model used derived from the same theoretical basis as adjuvant psychological therapy (Greer *et al.*, 1991) but the professional input of time was less. With first appointment lasting no more than 1 h and follow-ups no more than 30 mins, the mean total consultation time spent on each patient was about 3 h. With intervention from a fully trained clinical psychologist, Grade A at Spine Point 24 on the salary scale (and allowing 40% for employers' costs), the intervention cost approximately £45 per patient at current rates.

Ramirez (1989) questioned whether half of the patients referred to her liaison psychiatry service needed to see a psychiatrist. One-third of the patients in our audit had low scores on the HAD scale, and even among those with higher scores the question remains as to whether the same improvement could have been achieved more cost-effectively. Such an evaluation would need to take into account not only the immediate outcome but also the subsequent incidence of recurrent problems. It has been demonstrated in other settings (Blackburn *et al.*, 1986; Simons *et al.*, 1986) that there is a long-term advantage in favour of interventions which involve patients as active partners in learning strategies for coping not only with current problems but also with potential future difficulties.

The patients included in the survey were broadly representative of the workload of this regional cancer centre. The sample was biased only in that acutely or terminally ill and demented patients were excluded from the assessment procedure. Different assessment procedures, particularly including carers, are needed for this group. Only 3% of those approached refused to take part in this study. Twelve per cent who agreed to take part failed to complete the forms during their time in hospital or to return them by post afterwards. In general, the patient assessment procedure was feasible to administer and acceptable to the majority of patients seen in the department.

However, no staff support was offered to patients in completing the self-report measures employed, and the data show substantial numbers of missing items, particularly in the MAC scale, where 15% of the forms returned were incomplete. This underlines the point often made in QL assessment studies that the use of the self-report instruments still requires some investment of staff time to ensure the quality control of data collected. (Aaronson, 1992).

Doctors' ratings of anxiety and depression were significantly associated with patients' HAD scores in both samples, but in routine clinic practice it proved difficult reliably to obtain even a simple four-point rating of these two key dimensions for the purposes of this survey. Doctors were also aware of the scarcity of professional support services for patients. It is not surprising then to find that 30% of patients in the survey who warranted further assessment of their anxiety and 23% who warranted assessment for depression, i.e. HAD scores $\geqslant 8$, were not referred for help.

In the subset of the survey for whom more detailed doctors' ratings of problems were available, the proportions of patients recognised as having significant psychosocial concerns or difficulties was significantly different from patients' self-reports for all problems except the problems of relationship with hospital staff. Given that these ratings refer to in-patients and day patients, whom staff have more time to assess, it seems unlikely that the detection of patients' problems would be more accurate in the clinic.

The need for better screening for psychosocial concerns was demonstrated not only by the 99 surveyed patients who would have liked help but by the finding that it is not only severe problems but the cumulative impact of multiple problems which can undermine patients' coping resources.

Given that the problem checklist devised for this study proved easy to administer, acceptable to patients and capable of highlighting clinically relevant problems which are otherwise undetected, it would seem potentially useful as a means of promoting better communication and monitoring patients' psychosocial well-being, particularly in the out-patient setting. Completed by the patient in the waiting room, the checklist could alert the clinician to the specific problems requiring more assessment during consultation. Information from the checklist could readily be summarised on a single page in the case notes to prompt systematic enquiry and to monitor change over time. Severe, persistent and/or multiple problems could then be referred for more detailed assessment to an appropriate professional.

The specific concerns on this checklist evolved from clinical experience of patients referred to the clinical psychology service in the past. For particular settings additional problems may need to be monitored. The point emerges very strongly that, independent of the nature and severity of the specific concerns, having multiple ($>5$) problems was the best single predictor of distress. Clearly, there is value in a method which identifies the danger of overload when the component stressors may individually fail to register as being significant in degree.

The range of problems endorsed by patients in this survey underlines the need for oncology centres to provide an integrated range of supportive services to meet their patients' psychosocial needs. It is naive to think that any one professional group will have all the knowledge and skills to address the problems raised by cancer and its treatment. Methods of screening for potentially remediable psychosocial problems, doctors' referral practices and outcomes of different service provisions warrant more controlled investigation in order to determine the most cost-effective means of relieving the most common of cancer patients' concerns.

**Acknowledgements**

We should like to acknowledge the financial support of the Imperial Cancer Research Fund and the help of Nigel North, Julie Auchterlonie and Linda Gibson in data collection and management. We should also like to thank all the consultant staff of the NHS Departments of Clinical Oncology and Haematology and of the ICRF Medical Oncology unit for permission to include their patients in the study and most particularly for the help they and their staff gave with our assessment.

### References

AARONSON NK. (1992). Assessing the quality of life of patients in cancer clinical trials: common problems and common sense solutions. *Eur. J. Cancer*, **28A**, 1304–1307.

BLACKBURN IM, EUNSON KM AND BISHOP S. (1986). A two year naturalistic follow up of depressed patients treated with cognitive therapy, pharmacotherapy and a combination of both. *J. Affective Disorders*, **10**, 67–75.

CHRIST GH. (1991). A model for the development of psychosocial interventions. *Recent Results Cancer Res.*, **121**, 301–312.

CUZICK J. (1984). A Wilcoxon-type test for trend. *Stat. Med.*, **4**, 87–90.

FALLOWFIELD LJ. (1991). Counselling and communication in oncology. *Br. J. Cancer.*, **63**, 481–482.

GREER S, MOOREY S AND BARUCH J. (1991). Evaluation of adjuvant psychological therapy for clinically referred cancer patients. *Br. J. Cancer*, **63**, 257–260.

MAGUIRE P. (1985). Improving the detection of psychiatric problems in cancer patients. *Soc. Sci. Med.*, **20**, 8; 819–823.

OSOBA D. (1993). Self-rating symptom checklists: a simple method for recording and evaluating symptom control. *Cancer Treat. Rev.*, **19**, (Suppl. A), 43–51.

RAMIREZ AJ. (1989). Liaison psychiatry in a breast cancer unit. *J. R. Soc. Med.*, **82**, 15–17.

SCHAG CAC, GANZ PA AND HEINRICH RL. (1991). Cancer rehabilitation evaluation system – short form (Cares-SF). *Cancer*, **68**, 1406–1413.

SIMON AD, MURPHY GE, LAVINE JL AND WETZEL RD. (1986). Cognitive therapy and pharmacotherapy for depression: sustained improvement over one year. *Arch. Gen. Psychiatr.*, **43**, 43–49.

SLEVIN ML, PLANT H, LYNCH D, DRINKWATER J AND GREGORY WM. (1988). Who should measure quality of life, the doctor or the patient? *Br. J. Cancer*, **57**, 109–112.

WATSON M, YOUNG GJ, INAYAT C, BURGESS C AND ROBERTSON B. (1988). Development of a questionnaire measure of adjustment to cancer: the MAC scale. *Psychol. Med.*, **18**, 203–209.

WATSON M, GREER S AND BLISS JM. (1989). *Mental Adjustment to Cancer (MAC) Scale Users Manual* (available from the first author). Royal Marsden Hospital: Sutton, Surrey.

ZIGMOND AS AND SNAITH RP. (1983). The hospital anxiety and depression scale. *Acta Psychiatr. Scand.*, **67**, 361–370.